# Cellulose Nanomaterials—Binding Properties and Applications: A Review

**DOI:** 10.3390/molecules23102684

**Published:** 2018-10-18

**Authors:** Ali H. Tayeb, Ezatollah Amini, Shokoofeh Ghasemi, Mehdi Tajvidi

**Affiliations:** 1 School of Forest Resources, University of Maine, 5755 Nutting Hall, Orono, ME 04469, USA; ezatollah.amini@maine.edu (E.A.); shokoofeh.ghasemi@maine.edu (S.G.); mehdi.tajvidi@maine.edu (M.T.); 2 Advanced Structures and Composites Center, University of Maine, 35 Flagstaff Road, Orono, ME 04469, USA

**Keywords:** nanocellulose binders, cellulose nanofibrils, cellulose nanocrystals, biopolymeric binder, bacterial cellulose, particleboard, energy storage devices, paper coating, bone regeneration, biomedical

## Abstract

Cellulose nanomaterials (CNs) are of increasing interest due to their appealing inherent properties such as bio-degradability, high surface area, light weight, chirality and the ability to form effective hydrogen bonds across the cellulose chains or within other polymeric matrices. Extending CN self-assembly into multiphase polymer structures has led to useful end-results in a wide spectrum of products and countless innovative applications, for example, as reinforcing agent, emulsion stabilizer, barrier membrane and binder. In the current contribution, after a brief description of salient nanocellulose chemical structure features, its types and production methods, we move to recent advances in CN utilization as an ecofriendly binder in several disparate areas, namely formaldehyde-free hybrid composites and wood-based panels, papermaking/coating processes, and energy storage devices, as well as their potential applications in biomedical fields as a cost-effective and tissue-friendly binder for cartilage regeneration, wound healing and dental repair. The prospects of a wide range of hybrid materials that may be produced via nanocellulose is introduced in light of the unique behavior of cellulose once in nano dimensions. Furthermore, we implement some principles of colloidal and interfacial science to discuss the critical role of cellulose binding in the aforesaid fields. Even though the CN facets covered in this study by no means encompass the great amount of literature available, they may be regarded as the basis for future developments in the binder applications of these highly desirable materials.

## 1. Introduction

### Background and Motivation

Serious environmental concerns about the consumption of fossil-fuel based materials, such as green gas emissions, long degradation time, human health issues, etc., have forced technology to seek to employ sustainable nature-friendly materials as a replacement for petroleum-derived products. Among existing biopolymers, the straight-chain polysaccharide cellulose has long been recognized as the most abundant renewable organic polymer in the biosphere, having excellent mechanical characteristics, economic production and yet fairly functionalizable. Whether in the form of fibers or derivatives, cellulose constitutes nearly one trillion tons of the world’s annual biomass production [1], and to date, it has had a major impact on the development of numerous eco-friendly materials. Recently, there has been a resurgence in interest in utilizing cellulose to produce advanced materials and undoubtedly, it will play a critical role in the future of the (bio)economy. Cellulose is a fibrous water-loving polymer, essential in the structure of plant cell walls and naturally formed through the plant cellular development process and cellulose biogenesis, fixed by van der Waals forces and hydrogen bonds [2,3]. It is also found in some marine creatures (e.g., tunicates), fungi, invertebrates, and algae, as well as bacteria [4]. Two distinguished regions within cellulose fibrils are the crystalline and amorphous parts. By applying chemical processes, cellulose nanocrystals (CNCs) can be produced through the isolation of crystalline domains. Mechanical treatments on the other hand generate cellulose nanofibrils (CNFs) [3]. Lately, utilization of CNFs and CNCs with nanoscale lateral dimensions has garnered increasing attention, not only for their intrinsic biodegradability and abundance, but also due to unsurpassed quintessential properties, such as stiffness, low density, flexibility, large aspect ratio and unique rheology, which are, in part, due to their crystalline assembly via hydrogen bonds [3,5,6,7,8,9,10]. Also their high chemical functionality is due to the abundant primary and secondary hydroxyls (referred to as cellulose alcohols) on the surface, which can be easily modified with bio-polymers and yield cellulose derivatives or by grafting to different materials [11]. These OH groups can attract each other electrostatically (via hydrogen bonds), causing the chains to build an ordered structure [2]. Such hydrogen bonding throughout the polysaccharide has a key function in binder applications related to adhesion between cellulose nanoparticles and other materials [12], for example, in hybrid composite and paper production [13,14,15,16], environmental and water-related areas [17], biomedical applications [18], as well as energy storage devices [19,20].

In the present work, first we briefly introduce the structures, types and pertinent production methods of cellulose nanomaterials and then review some of the recent studies on CN-based binders in four separate areas, namely hybrid composites and wood panels (in light of recent efforts to replace common carcinogenic industry adhesive ingredients, particularly formaldehyde, by CNs), papermaking/coating processes, energy storage devices and finally, the potential usage of nanocellulose in biomedical fields as a cost-effective and tissue-friendly binder for cartilage regeneration, wound healing and dental repair. The key properties of CN for the intended binding applications will be delineated separately in the corresponding sections but overall their bio- and tissue-friendly nature, high aspect ratio, excellent mechanical attributes and ability to establish extensive H bonds within various polymeric matrices are among the most important features. Also relevant to its binding performance, they are capable of building a wide range of CN-based hybrids via self- or directed-assembly [3,10,13,21,22].

## 2. Chemistry of Nanocellulose and Its Production Methods

Cellulose is a linear-chain homopolymer composed of repeating ringed anhydro-d-glucose monomers with the general formula of (C_6_H_10_O_5_)_n_, (n < 20,000). Each repeating unit is rotated 180 degrees around the axis of the cellulose backbone relative to the neighboring ring, linking together via a β-(1,4)-glyosidic bond [21,23,24]; the C1 atom of the first ring is attached to the C4 one of an adjacent glucose through a covalent oxygen bond.

**Figure 1:**
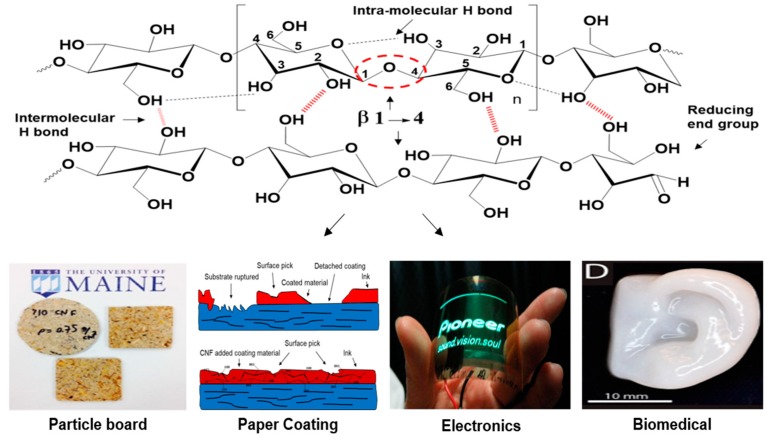
Cellulose structure and intra/intermolecular hydrogen bonding pattern (**top**). Example images summarizing the topical focus of current review, wherein the recent development of nanocellulose materials and their binding-related applications in particle boards, paper coating, electronic devices and biomedical science (e.g., in cartilage regeneration) is covered (**bottom**). Images labeled as “Electronics” and “Biomedical” are reprinted with permission from [26]. Copyright 2009 Elsevier and [27]. Copyright 2015 American Chemical Society, respectively.

As illustrated in Figure 1, the electrostatic attractions between oxygen and hydrogen atoms of the adjacent rings, inducing intra-molecular hydrogen bonding, cause more stabilized glycosidic linkage motifs, linear-chain configuration, in addition to feeble solubility in polar solvents [24]. Also, inter-chain hydrogen bonds (occurring between polymer chains), along with van der Waals forces, promote parallel stacking, known as cellulose elementary fibrils, which can further aggregate into micron-scale predecessors [24]. These intra- and inter-chain non-covalent attractions are vital for the stability and firm structure of cellulose, the latter of which is essential for plants and some marine creatures. Depending on the primary and secondary alcohols on the surface; C2-OH, C3-OH and C6-H_2_OH, cellulose may have different degrees of hydrogen bonding. For example, regenerated cellulose (cellulose II) which is produced via a mercerization process, demonstrates altered hydrogen bonding motifs, with a stronger and more solid structure compared to natural cellulose (cellulose I) [25].

Within cellulose fibrils, there are two discrete domains: highly ordered (crystalline) and disordered (amorphous) structures. By applying different techniques, two commonly used forms of nanocellulose, cellulose nanocrystals (CNCs) and cellulose nanofibrils (CNFs) can be separated from the cellulosic source. A third form, bacterial cellulose (BC) is synthesized by some Gram-negative bacterial species and differs from the two other types in that it is naturally formed by a bottom-up process as opposed to the top-down approaches used to produce CNC and CNF [7]. Collectively, all types of cellulose nanomaterials can be called cellulose nanoparticles (CNs). In addition to their unique physiochemical features (which will be discussed later in this review), CNs have high potential as substituents for synthetic petroleum-based binders and adhesives. In the next section, we focus on the preparation techniques along with the properties of each group.

### 2.1. Cellulose Nanofibrils (CNFs)

CNFs are nano-scale fibrils with high aspect ratio; their width varies from 2 to 60 nm (being several micrometers in length) and are formed as a result of cellulose chain-stacking, induced by hydrogen bonds [28]. These nanoparticles (also known as nanofibrillated cellulose, NFC, or microfibrillated cellulose, MFC) which consist of both crystalline and amorphous domains, can be produced by liberating the fibrils from the integral microfiber bundles through vigorous mechanical fibrillation processes [21,29]. By far, many reviews have discussed their exceptional characteristics, such as biodegradability, excellent mechanical properties, high surface area, light weight, etc. [3,5,6,21,30]. The hydrogen bonds between the hydroxyl groups prompt a highly ordered conformation of the cellulose chains in elementary fibrils that further facilitate adhesion with other polymeric components, e.g., with lignin or proteins, to form networks in aqueous media [12,31]. Since an energy-intensive mechanical grinding is required to separate the stacked fibrils, different types of pretreatments, such as alkaline [32,33], radiation [32], chemical [34,35], and enzymatic [36,37] are typically applied prior to the fibrillation process to remarkably lower the cost and energy. For this reason, CNF manufacturing at an industrial scale was not feasible until recently [38]. A production technique which has been used frequently is pre-oxidation using (2,2,6,6,-tetramethylpiperidin-1-yl) oxidanyl, abbreviated as TEMPO, to reduce the grinding cycles prior to applying the vigorous mechanical fibrillation by microfluidization or homogenization. The resulting nanoparticles give a gel-like appearance at low solid concentrations (1–2%) in water [38,39], and once formed into a transparent film, display high mechanical integrity, limited oxygen transmission rate as well as low thermal expansion (~2.7 ppm K^−1^. m) [39,40,41]. Translucent thin films from microfibrillated cellulose (20–100 nm in diameter) were also introduced by Okamura et al. [42]. So far, researchers have used CNF as reinforcing agents in different composites such as hybrid plastics, papers and thin packaging films. For instance, the use of CNF in the papermaking wet-end results in papers with higher strength [43].

### 2.2. Cellulose Nanocrystals (CNCs)

Whisker-shaped CNCs, (also named cellulose nanowhiskers), are reminiscent of the crystalline regions within elementary nanofibrils of cellulose during the biosynthesis process. They are isolated from the cellulose amorphous domains of wood/plant fiber, microfibrillated or nanofibrillated cellulose [44,45,46]. The isolation procedure is based on an acidic attack through transverse hydrolysis along the fibrils disordered regions, leaving highly crystalline domains that resist acid hydrolysis [21,47].

**Figure 2:**
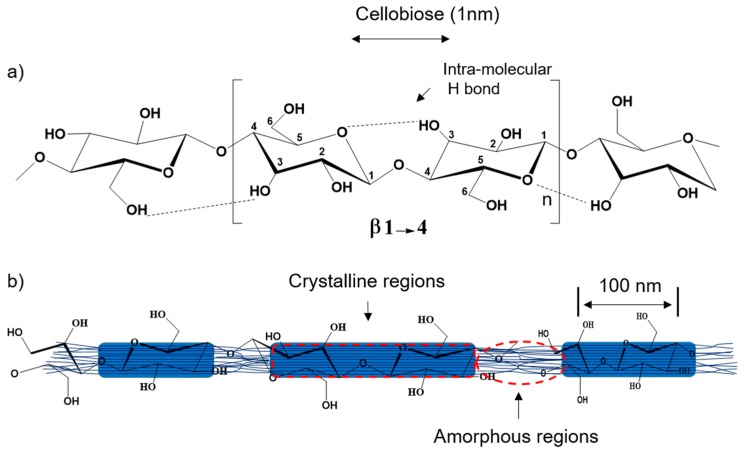
(**a**) Schematic of cellulose repeating unit with the β-(1,4)-glycosidic linkage, dotted lines indicate intramolecular hydrogen bond; (**b**) hypothetical configuration of ordered (crystalline) and disordered (amorphous) regions in cellulose nanofibrils.

In Figure 2, these ordered areas are due to the linear nature of the cellulose polymers and the extensive intermolecular attractions between adjacent chains [30]. The final products, that contain crystalline fragments, resemble whiskers or rods, owing to their end tapering that has occurred during the cleavage process.

**Figure 3:**
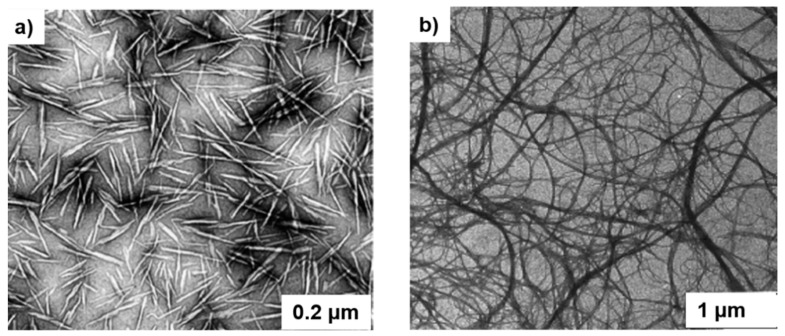
TEM images of (**a**) dried dispersion of cellulose nanocrystals (CNC) derived from ramie, (reprinted with permission from [49]. Copyright 2008 Royal Chemical Society); (**b**) dispersion of nanofibrillated cellulose, CNF (reprinted with permission from [50]. Copyright 1997 John Wiley & Sons).

Figure 3 shows the morphological differences between CNF and CNC. Compared to CNFs, cellulose nanocrystals have lower aspect ratios (100–500 nm in length and 10–50 nm in width) and depending on the process condition, a high degree of crystallinity (50–90%) [30,48]. Studies have been conducted on different cellulosic sources to optimize the CNC isolation for better quality and production yield [21,48].

Among the various acids, hydrochloric and sulfuric acid have been used extensively for this purpose [51,52]. During the hydrolysis by sulfuric acid, negatively charged sulfate groups will be introduced on the cellulose chain through esterification of hydroxyls. These groups boost intermolecular repulsive forces, causing electrostatic stability of CNCs in polar aqueous suspensions [51]. While pristine cellulose has little reactivity, CNCs can easily be modified to form numerous derivatives and impart physiochemical features such as transparency, stiffness, low density and inducing tunable surface chemistry [9]. Once the concentration of these crystalline needles exceed a critical level, they form ordered, birefringent phases in colloidal systems [53]. A number of studies, have been conducted on producing nanocrystalline cellulose and its application in a variety of fields such as hybrid composites [54], barrier films, electronic devices [55], antimicrobial films, emulsion stabilizers, etc. [3,7,56,57]. For example, because of their high stiffness and surface area [58], as well as low roughness (~2 nm) and density (1.6 g/cm^3^) [4], CNCs have attracted attention for use in flexible substrates [59]. They also possess excellent Young’s moduli of around 150 GPa that make them a suitable candidate as green reinforcing materials in composites [23,60,61].

### 2.3. Bacterial Cellulose (BC)

**Figure 4:**
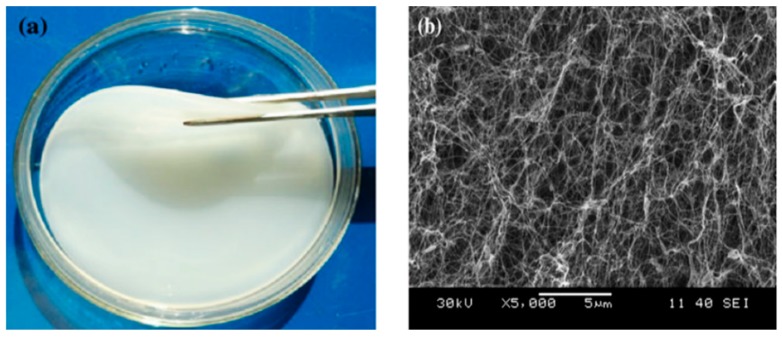
(**a**) Produced bacterial cellulose in static cultivation; (**b**) scanning electron microscope image of a BC network (reprinted with permission from [66]. Copyright 2014 Springer).

Another group of cellulose nanoparticles differing from the fiber-like CNF and needle-shape CNC is bacterial cellulose (also referred to as microbial nanocellulose, or biocellulose). Bacterial cellulose (BC) contains ribbon-shape nanofibers linked together through a network of hydrogen bonds. It is formed by various microorganisms and bacterial species (e.g., *Acetobacter xylinum, Pseudomonas*) [62], that are found where carbohydrate fermentations take place [63,64,65]. Unlike other nanocelluloses, BC is produced through a bio-fabrication assembly process from low-molecular weight sugars such as d-glucose and is isolated as an exopolysaccharide at the air-nutrient media interface [66]. Therefore understanding its biosynthetic process is important to better tune the culturing condition and alter the desired nanofibrils structure and crystallinity which may lead to new properties, functionalities, and applications for such bacterial-derived nanocelluloses [67]. Figure 4 shows a hydrogel sheet of bacterial cellulose (both its 3D structure and a microscopic image) that was produced via a static cultivation method.

Despite having similarities to CNF and CNC, the nanofibril bundles in BC have some unique properties such as more crystallinity, higher degree of polymerization (up to 8000), as well as ability to form an extremely ultrafine web structure, that makes it difficult to disperse in water [64,68,69]. BC also has fibers with a high elastic modulus of 78 GPa [70], (close to that of glass fibers, and greater than natural nanoscale fibers), and possesses a larger water holding capacity compared to plant- derived cellulose [71]. Additionally, the light weight, biodegradable and non-toxic bacterial cellulose is produced as pure cellulose and avoids the traditional chemical treatments required for lignin or hemicellulose elimination [9,72].

Several studies have indicated the possible usage of bacterial cellulose as a strengthening agent, transparent film and binder in hybrid nanocomposites [73,74,75,76]. Lee et al. made a robust sisal fiber preform using bacterial cellulose as binder and showed the resulting composite had better performance and mechanical properties compared to a commonly used polymer poly (acrylated-epoxidised soybean oil-based polymers (poly-AESO) [77]. Also the combination of BC and conducting polymers (e.g., polyaniline, polypyrrole) has resulted in a fully biodegradable inter-connected structure, suitable for aerogels and transparent composites [78].

## 3. Nanocellulose as Coating Material

### 3.1. Traditional Paper Coatings

The potential of using CNF in paper coatings has been intensely studied for the formation of a continuous layer on paper surfaces to induce suitable qualities such as mechanical strength, fire retardancy and water/gas impermeability [79,80,81,82,83,84,85]. Because the properties of coated surfaces depend highly on the CNF distribution and the corresponding layer thickness, an appropriate coating formulation is critical to achieve the desired features. CNF was applied to a paper surface via two different methods: roll coating and size press, resulting in coat weights of 14 and 3 g/m^2^, respectively [86]. A thin, well spread layer of CNF can also be attained by spray coating. The possible challenge however is the preparation of low solid content solution that may both affect the drying process and/or lead to breakage of the web [86]. Another issue is the high viscosity of the CNF suspension, even at low solids, and the absorption of water by the base paper which can lead to localized thickening of the coating material and create uneven spreading. A recent work has shown that the use of only 4% carboxymethyl cellulose (CMC) based on the dry weight of the CNF can lead to a substantial drop in viscosity and better coverage of the paper surface [87]. CNF has been also coated on paper via foam coating a mixture of 2.9% CNF and an anionic surfactant under 80–95% compressed air. The resulting double-layer had a coat weight of 1–2.6 g/m^2^ [88]. Others meanwhile have reported the application of CNF at low solids and moderate speeds, and lately it has been shown possible to apply CNF at high speeds (10 m/s) and moderate solids (up to 5%) using a cylindrical laboratory coater (CLC). Coat weights in double layers as high as 10 g/m^2^ were reported [89].

### 3.2. CNF as Binder in Coating Layers

**Figure 5:**
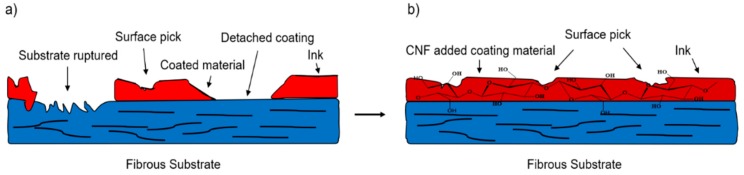
Schematic of hypothetical CNF effect to improve the coating material bonding: (**a**) the defective surface with no CNF; (**b**) same surface with CNF incorporation.

The application of CNF as binder in conventional paper coating processes is a relatively new concept that has garnered a great deal of attention. Owing to its strong hydrogen bonding and the ability to establish a uniform layer on hydrophilic substrates, CNF was suggested as a green binder to improve the properties of coating layers on paper/paperboard and reduce the level of latex [15,86,88,90,91,92]. Figure 5 presents the hypothetical positive role of CNF in the coating process in which it helps the coating material to bind better on the fibrous surface and form a less defective and smoother surface. A low ratio of CNF was applied to attach clay onto paper surfaces and the obtained CNF-clay layer produced a significant raise in surface smoothness and print density, almost identical to when polyvinyl alcohol (PVA) was used as binder [91]. In another study, a CNF-latex mixture was employed as a potential binder in color coating and it was revealed that the presence of CNF increased the picking strength while slightly reducing the paper surface energy [90].

To address this issue other methods such as inkjet or flexographic printing can be used where CNF is employed as a binder in coating formulas. Enzymatically pre-treated CNF and TEMPO- oxidized CNF were investigated as alternative binders (for partial latex replacement) to bind calcium carbonate particles in coating formulations. The results indicated a successful replacement of 20% of latex with CNF in coating colors with no evidence of printing or runability issues [92]. Nevertheless, an adverse effect on the energy consumption during the drying process was seen and attributed to the higher water content of the coating layers. A pertinent study indicated a blend of CNF and starch could significantly improve the coating layer properties and produce a low-linting surface, indicating the role of CNF to link the starch molecules onto the paper surface. In conventional coating processes, different coat weights of 8–12 and 0.5–2 g/m^2^ are used for pigment and starch, respectively [86]. CNFs have also been utilized to bind graphite to cellulose nanocomposites [93]. Also, addition of CNF in paper coating could increase the printing pick resistance, improve the strengths and modifying the rheology of the coated layer [10,94]. However, a key issue for using CNF in the coating process is its rheology, that even at low solid content (<2%) gives a viscous suspension that hardly loses its water [95].

To better understand the role of CNF as a binder in coating processes, knowledge of the possible interactions between nanocellulose and paper substrate is essential. It is known that cellulose to cellulose adhesion takes place from molecular to several millimeter scales (Table 1). Hydrogen bonds and van der Waals forces are considered the dominant intermolecular forces contributing to the adhesion strength, yet it is not fully understood which one is of central importance to the binding strength [16]. Also physical entanglement that relies on mechanical fibrillation (within micron scale) can boost cellulose-cellulose bonding in paper and paperboards to a great extent. This phenomenon occurs during the beating process used to promote the fiber interlocking phenomena [96].

### 3.3. Superhydrophobicity Induced by CNF

Superhydrophobicity is a fundamental attribute of solid surfaces that plays an essential role in many household and industrial applications. By definition, any surface with a water contact angle greater than 150 degrees is considered as superhydrophobic [99]. Among many substrates, such as plastic, glass, metals, etc. producing water-repelling cellulose, e.g., hydrophobic paper, has attracted a great deal of attention owing to its cost effectiveness, wide availability and in part for the recent impetus of environmental conscience [100]. Therefore, fast-growing efforts to produce hydrophobic bio-based substrates, such as papers, are being reported more often.

Some of the common methods to fabricate hydrophobic papers are plasma etching [101,102], ink-jet printing [103], grafting hydrophobic molecules [104] as well as deposition of nano-materials [105], or hydrophobic chemicals such as alkyl ketene dimer (AKD) [106]. Ondra et al. [107] created an extremely water repellent surface (water contact angel of 174°) by applying AKD to a glass surface, despite the fact that the intrinsic contact angle of smooth AKD has been reported as 109°. They ascribed this extra hydrophobicity to the fractal growth of AKD crystals, leading to formation of a micro-size highly rough surface [107]. Another approach is to use precipitated calcium carbonate (PCC) to provide such a micro-scale roughness for the subsequent AKD deposition [108,109]. However PCC particles form loose attachments to the paper surface and additional chemicals are necessary to adhere them to fibers. To tackle such issue, CNF was suggested as a binder to achieve the desired hydrophobicity on the paper [15,110]. In a study by Arbatan et al. CNF was applied to link PCC pigments onto the paper surface, providing the required rough surface prior to the sizing level. In that work, a dip-coating approach was used to form a PCC layer and then AKD was applied to achieve a superhydrophobic surface [110]. A related study showed the positive effect of CNF to link latex on paper surfaces during the coating process, however, its presence reduced the paper surface energy to some degree [90]. It is known that two key factors to obtain ultra-water repellency are the low surface energy plus nano-roughness [111] and reviewed works in this section provide evidence that using CNF as a binder may contribute to both these aspects. The cited work regarding the PCC attachment to paper via CNF was an example of change in surface morphology whereas in the case of CNF-latex mixture, it could alter the surface free energy. Despite major advances in obtaining low/ultra-low energy interfaces from polymeric materials, there are still limited studies on the case of nanocellulose. Such an approach, if proved successful, can highly benefit packaging and food storage industries in which water barrier properties are at the center of interest.

## 4. Nanocellulose in Energy Storage Devices

There is a high demand for low-cost green energy storage devices, with large energy density, recyclability and a harmless core disposal. To achieve these goals, employment of biodegradable polymers such as cellulose has been suggested to be adopted in batteries as electrode binder, battery body and even electrolyte material [112]. Electrode binders should be electrochemically stable and are used to attach active materials to the composite electrode. They can affect the irreversible capacity losses and provide stability to electrode cycles by withstanding dimensional changes during charging-discharging periods while keeping acceptable energy density [112,113,114]. Another important aspect of binders is their solubility. Polyvinylidene difluoride (PVDF) is a common strong electrode binder, however it is only soluble in nonpolar solvents which are mostly flammable, expensive and pollutant. Replacing such material with water-soluble ones, can both reduce the environmental impact and production cost [115]. Here we only focus on the binder application of nanocellulose in energy storage devices.

### 4.1. Nanocellulose as Flexible Electrode Binder

CNF, is a renewable polymer and can self-assemble into a continuous film with high stiffness, transparency, flexibility and low thermal expansion [6,7,116]. Therefore there is a great potential for CNF utilization as a binder in energy storage devices (e.g., electrodes in Li-ion batteries), alternative to petroleum-based polymers [117,118,119]. Here, some of the recent advancements in the development of CNF-based binders in battery components are reviewed.

In a study on lithium ion batteries, electrolyte saturated paper and dispersed nanofibrillated cellulose were used as separator and electrode binder, respectively. The achieved binding quality was similar to PVDF [120]. Similarly, a CNF network (as binder) was placed around graphite platelets via a simple water-based casting method to induce porosity, flexibility and high charging capacity to graphite anode. The resulting self-standing electrodes showed a great electrochemical performance, close to that of conventional PVDF-based electrodes [93].

**Figure 6:**
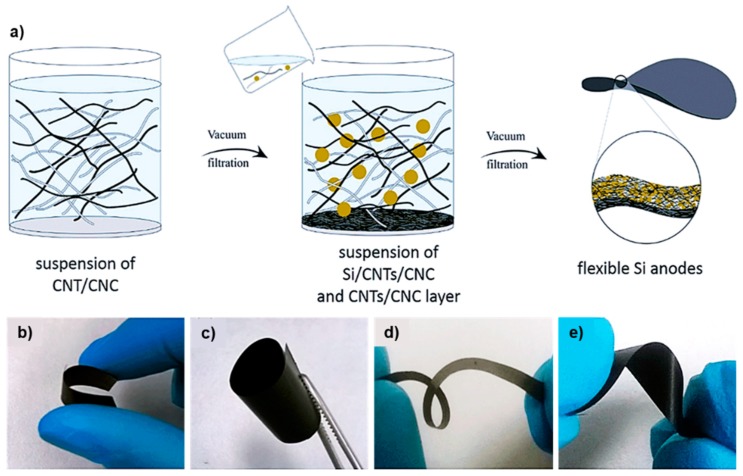
(**a**) Schematic visually summarizes the manufacture process of the flexible Si-CNT-CNC composite electrode via filtration process. Photographs of the respective electrodes under bending (**b**) rolling (**c**) and twisting (**d**–**e**). Reprinted with permission from [123]. Copyright 2015 Royal Chemical Society.

An MFC-polypyrrole (PPy) electrode composite was made through coating microfibrillated cellulose on polypyrrole (via in situ polymerization) to obtain self-standing conductive paper for a more ecofriendly binder system [118]. Also, an environmentally-benign flexible battery cell has been suggested through incorporation of CNF into a single paper layer to couple into the electrode active materials. The process involved a sequential water filtration and the attained films (250 µm in thickness) displayed acceptable cycling performance upon prolonged testing [121]. In a different study, CNF was used in cathodes formulation made of LiFePO_4_-carbon black-CNF via filtration technique and the resulting cellulose-based electrodes demonstrated a high Young’s modulus and discharge capacity of 100 MPa and 110 mAhg^−1^, respectively [122]. Likewise, as shown in Figure 6, a freestanding flexible Si/CNC-based anode was produced in a ternary system consisting of nanocellulose, carbon nanotubes and silicon nanoparticles as building blocks. The strong attachment of Si particles to the porous nanocellulose-carbon nanotubes matrix resulted in a high specific capacitance and excellent charge-discharge cycling performance applicable for lithium-ion batteries.

Besides, no adverse impact on film’s flexibility and tensile properties were observed upon incorporation of Si nanoparticles into the CNC-CNT matrix. The attained anode could be easily rolled and twisted with no performance loss (Figure 6b–e) [123].

However, one challenge in such applications is the amount of water trapped in the nanofibrils. Only low-loads of CNF (~4 wt %) could be used to avoid possible adverse effect on the performance, while improving the corresponding energy capacity. Water content value, more than 20−50 ppm, can lead to lithium salt degradation and since cellulose may contain from 20,000 to 120,000 ppm of trapped water, applying a prolonged thermal treatment is necessary to remove the excess water [121,122,124,125]. Besides, in spite of promising adhering effect of CNF, it has a relatively low pliability around the electrode which can restrict its utilization in flexible devices [126,127]. For this, modified nanocelluloses such as TEMPO-oxidized CNF have been proposed owing to their potent structure, flexibility and compatibility with water-based systems [128]. Moreover, cellulose treatment with ionic liquids may be used in flexible electronic devices when natural CNF is not the ideal choice. One study reported that bendable CNF films can be produced for electrochemical double layer capacitors once they are treated with high voltage ionic liquid electrolytes [129]. Further investigation is necessary to fully replace the current electrode binders with nanocellulose or its nano-scale derivatives, but if proved feasible, such alternative may fundamentally improve energy efficiency.

### 4.2. CNF Substrate for Supercapacitors

Supercapacitors are short-term electrochemical energy storages that are used in devices with rapid charge/discharge cycles at the electrolyte-electrode interface. They usually can tolerate a vast range of operational thermal stresses [130] and depending on the storage mechanism, they are divided into electrical double-layer capacitors (EDLC) and electrochemical pseudocapacitors [131]. Many efforts have been made to develop nontoxic, lightweight and bendable supercapacitors for usage in portable and stretchable electronics. To that end, materials such as polydimethylsiloxane (PDMS), graphene paper and cellulose have been presented as substitutions of commercial EDLCs [132,133,134,135,136].

Among capacitor components, binders have the vital role of providing enough mechanical strength to the electrodes. Polyvinyl alcohol (PVA), carboxymethyl cellulose (CMC) and polytetrafluoroethylene (PTFE) are common binder agents that have been used in commercial supercapacitors, however due to non-conductivity, they may lower the electrode efficiency [137]. Alternatively, various studies have proposed CNF in such devices to enhance the capacitors strengths and electrical properties. For instance, composite electrodes were fabricated using CNF and conductive polymers such as polyaniline and polypyrrole; a silver-coated CNF aerogel was utilized as a supercapacitor substrate and was electrodeposited by polyaniline (PANI). Upon air drying, the capacity of resulting assembly was monitored and a specific capacitance of 176 mF. cm^−2^ along with significant resistance to bending was reported [138]. In a similar study, a highly porous electrode substrate was obtained using cellulose nanocrystals film coated by polypyrrole. The produced capacitor showed excellent capacitance (mass normalized) of 256 F. g^−1^ owing to CNC-based porous structure and had better cycling function and durability compared to carbon nanotube [139]. Andres et al. have shown that CNF is significantly effective to affix graphite electrodes to the electronic substrates once soaked in aqueous electrolytes [140]. In another work, a bio-friendly ionogel was produced through gelation of microcellulose thin films with various methylphosphonate ionic liquids. The attained flexible ionogel acted as high capacitance dielectrics under low voltage which is applicable in paper-based supercapacitors [141]. Overall, nanocelluloses have been successfully employed in capacitors as binder and potentially can induce both strength and flexibility to the electronic devices.

## 5. Adhesion in Nanocellulose

For a long time the inherent self-adhesion of cellulose fibers has been recognized as a key factor in the strength properties of paper and other cellulose-based composite materials. The formation of hydrogen bonds between hydroxyl groups on neighboring cellulose surfaces plays a major role in the superb adhesion properties between cellulose fibers and makes CNF an excellent binder material for nanocomposite applications [142]. Hydrogen bonds on the cellulosic surfaces are the dominant factors that need to be managed in order to tailor the compatibility of nanocellulose with other materials. They also seem to have a crucial role in the partial irreversible pore closure within the cell wall structure of cellulosic fibers upon drying. This phenomenon that is called “hornification”, is essential for the formation of a strong and integrated structure in paper-based films [16,143]. However, the influence of other factors on unions between fibers has been confirmed as well.

Understanding the molecular level interactions between cellulosic surfaces is necessary to better control the attractive forces responsible for fiber to fiber surface linkages. Polar acid-base interactions and van der Waals dispersion forces are important factors in CNF surface adhesion. Depending on the cellulosic sources and the preparation method, the resulting interfaces may display different surface free energies. While certain types of cellulose can show a predominantly polar behavior, others may have higher dispersion characteristics [12]. The polar chemistry of nanocellulose reduces its affinity to lipophilic components to a great extent [144]. Also degree of crystallinity may affect the cellulose binding ability as well; less ordered regions within CNF chains can lead to higher accessibility of the functional groups on the surface and result in better interfacial adhesion [145].

## 6. CNF as Binder in Particleboard, Fiberboard and Laminates

One of the most promising binder applications of CNF is in the production of particleboards and medium density fiberboards, as a replacement for urea-formaldehyde (UF) resin. An important downside of UF-containing boards is the emission during both manufacture and use of formaldehyde, whose carcinogenicity has been proved [146]. Cellulose nanofibrils are able to establish strong bonding between wood particles/fibers through a formaldehyde-free manufacturing process and provide an all-sustainable alternative for these systems as they are entirely independent of petroleum-based chemicals. CNF can form into excellent flexible and strong films by a simple drying process [9]. At low thicknesses (on micrometer scale), this structure can nearly be considered as plane isotropic due to the high aspect ratio of CNFs and their tendency to lay flat randomly in the x-y plane. Given that at low solids content, CNFs can be well dispersed in water and form a hydrogel, a three-dimensional network of fibrils that holds the particles together upon the drying process can be speculated. Considering the exceptionally high mechanical properties of cellulose nanoparticles [147] and their ability to establish hydrogen bonds within themselves and with other cellulosic materials [148], they can be suggested as binder in other lignocellulosic materials including wood particles, pulp fibers and paper sheets to induce strongly bonded composite systems.

**Figure 7:**
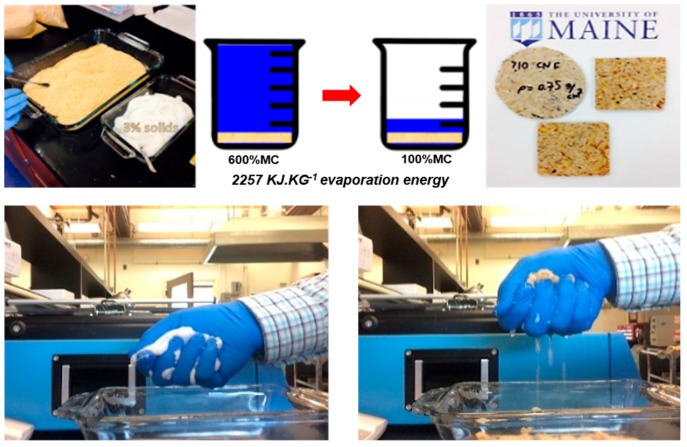
(**Top**) raw materials (**left**) to produce CNF bound particleboard (**right**). A 500% reduction in the initial moisture content is achieved without using a source of heat leading to significant energy savings (**center**). Initial samples made (**right**). (**Bottom**) contact dewatering of CNF in the presence of wood particles.

At micro/nano scale it is expected that smaller particles of CNF can penetrate into the porous structure of wood particles or other lignocellulosic materials and provide stronger bonds. An inter-diffusion process can be hypothesized when CNF dries in contact with the rough and porous surface of wood particles in the panel. Figure 7 shows preliminary samples made by mixing/pressing Southern Pine wood particles with a 3 wt % CNF suspension. At 15 wt % CNF content, the mixture to be pressed contains over 600% moisture content (based on dry weight of the wood particles) making it impossible to hot press because evaporating this large quantity of water may cause burst and delamination issues. However, it has been observed that most of the water in the mixture can be extracted via a simple cold pressing operation. Figure 7 also shows what can be termed “contact dewatering”, an interesting property of cellulose nanomaterials when they come in contact with other lignocellulosic materials. It is extremely difficult to squeeze out the water from a 3% solids content CNF suspension however once mixed with wood particles, even a mild squashing can result in water separation as seen in the bottom row of Figure 7. This reduces the moisture content of the mat to around 100% (50% solids) that is manageable by controlling the hot press cycle. Nevertheless it is still well above the average moisture content of a particleboard mat to be pressed in a conventional urea-formaldehyde resin system (around 8–10%). This points out to the necessity of more studies on dewatering and drying to accomplish lower press cycles.

**Figure 8:**
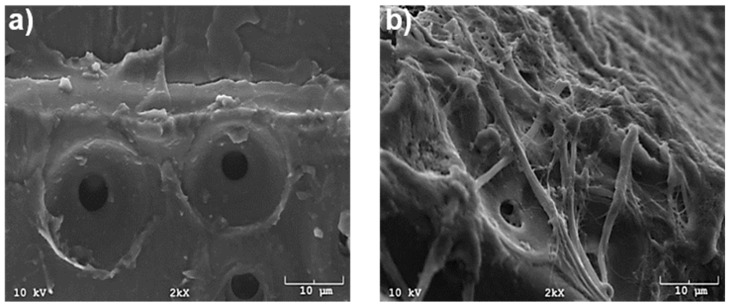
SEM image of a wood particle surface (**a**) and a particle mixed with a 3% solids content CNF slurry after air drying (**b**).

Figure 8 demonstrates how wood particles can be bonded together by CNF. CNFs are observed being distributed over the particle surface, some agglomerated into platelet shapes and some preserving their fibrillar morphology. It is speculated that at least smaller parts of the CNF particles in the suspension can penetrate into the pores/void spaces within the structure of wood particles to form a three-dimensional network of nanofibrils upon applying the hot press. The resultant fibrillar network can encompass the particles, bestowing higher strength and stiffness to the panel. The strength of bonds formed between the two wood particles is thought to be dependent on the degree to which interpenetration of CNF is occurred as well as surface characteristics of wood particles and nanofibrils that can control the extent of hydrogen bonding between them.

Efforts have been made to utilize CNF as an adhesive binder in the formulation of particleboards and medium density fiberboards. Kojima et al., in two separate studies, explored the possibility of mixing CNF and lingocellulose nanofibers (LCNF) with wood flour. They found that the mechanical and physical properties of the produced wood flour boards were improved with the addition of CNF and LCNF attributed to the occurrence of strong links between wood flour particles [149,150]. In another work, they reported significant improvements in the flexural, internal bond, and water sorption properties of the wood flour boards with inclusion of LCNF [151]. Feasibility of using cellulose nanofibrils as the sole binder in a “no-added formaldehyde binding process” for particleboard manufacture was first introduced by University of Maine [152], and later was briefly discussed in the work by Tajvidi et al. [15]*.* Amini et al., published a set of data and analysis of the mechanical and physical characteristics of wet-formed particleboards bonded only with CNF [14]. The mechanical properties of the produced particleboard panels were good enough to meet the industrial requirements for low density grades (below 0.64 g.cm^−3^). They also made an attempt to understand the adhesion mechanisms as well as the strength development involved in a wood particle-CNF system.

Effects of board density, CNF ratio, pressing method (constant pressure vs. constant thickness), and particle size on the flexural properties, vertical density profile (VDP) and internal bond (IB) strength of CNF-containing particleboards were investigated [153,154]. Density was shown to have the most significant effect on the modulus of elasticity (MOE), while the CNF ratio had the major effect on the modulus of rupture (MOR). The results also revealed that the press regime had significant effects on both VDP and IB strength. Panels produced by constant pressure (CP) and constant thickness (CT) pressing methods demonstrated flat-shaped and U-shaped density profiles, respectively. Higher IB values were observed for CP pressed panels compared to CT ones. The density and CNF ratio also influenced the IB strength and the VDP of the panels. They also studied [155], the effects of density, CNF level, particle size, and press program upon the nail and face screw withdrawal strength, water absorption (WA), and thickness swelling (TS). Increases in density and CNF level positively influenced the nail and face withdrawal strength. Better WA and TS performances were attained for CT pressed panels with smaller wood particles.

One major consideration in using CNF as binder in conventional boards is cost reduction. In this respect a viable path seems to be the use of lignocellulose nanofibrils (LCNF) in place of CNF. It has been shown that LCNF films are slightly weaker than CNF but the properties can be considerably enhanced when they are hybridized with CNF [156].

LCNF isolated from thermomechanical pulp (TMP) via atmospheric refining was used as adhesive replacement in fiberboard and the physico-mechanical properties of the resulting boards were evaluated at different LCNF contents and press temperatures [157,158]. The optimal processing condition was at 180 °C press temperature and 20% LCNF content, at which a maximum modulus of rupture of 12.1 MPa as well as a modulus of elasticity of 1.5 GPa were obtained for the produced fiberboards. By increasing LCNF content, a positive linear relationship between modulus of rupture (MOR) and internal bond strength (IB) was perceptible, regardless of the press temperature. Overall, LCNF-added medium density fiberboards acted well in the fulfillment of the commercial standards in terms of physico-mechanical attributes.

Cellulose nanofibers have been also used to improve the properties of conventional adhesives and binders for wood-based composite panels. Veigel et al. [159], investigated the impact of adding CNFs to urea-formaldehyde (UF) and melamine-urea-formaldehyde (MUF) resins on the flexural strength, internal bond, fracture energy as well as fracture toughness of the particleboard and oriented strand board (OSB). They found that, at a given resin consistency, adding a small amount of CNF will increase the viscosity of resin and decrease its spreadability which limits the addition level of nanocellulose. Adding 1 wt % of CNF (on a dry basis) resulted in enhancing the mechanical properties of particleboard and OSB panels with a more promising performance in OSBs. Also introduction of nanocellulose to the tannin-based adhesives yielded to higher mechanical behavior in particleboards [160]. Adding 2 wt % of CNF caused the viscosity of lignin-based adhesive to increase from 350 to 5462 mPa·s and the internal bond strength of the corresponding particleboards to increase by approximately 15%.

A novel laminate system comprising of sheets of paper bound together using CNF was reported by Shivyari et al. Bonding properties of CNF were evaluated through a series of peeling tests. Multi-layer composite laminates were produced from sheets of paper bonded together via CNF and the effects of nanocellulose solids content, press time/temperature on the physical and mechanical properties of the laminates were evaluated. Elastic modulus and strength of the laminates met or exceeded those of a short glass fiber reinforced polypropylene and various natural fiber-filled polypropylene composites and a number of wood and paper-based laminates [161].

## 7. Nanocellulose as a Binder for Natural Fibers

Recently nanocelluloses have been used for reinforcing other natural fibers taking advantage of their binder properties. Ghasemi et al. [162] studied the effect of CNF and CNC to strengthen natural fiber tape and yarns. They soaked hemp and flax fiber in nanocellulose suspensions and produced tapes and yarns by laying the fibers flat or spinning them, respectively. Improvements in tensile properties of tapes and yarns were reported and it was found that while CNC had a better performance on yarns mechanical properties, CNF was more effective on tapes. No relationship between the mechanical properties of yarns and those of the single fibers coated with nanocellulose was observed, indicating that enhancements in the mechanical properties of yarns were mainly due to nanocellulose role in improving bonding between natural fibers.

In another work, Pommet et al. [163], introduced a method in which they deposited bacterial cellulose (BC) on hemp and sisal natural fibers for improving their adhesion to bio-based polymers. While untreated fibers showed a poor adhesion with bio-based polymers, deposition of BC was promising to enhance surface adhesion of the fibers with cellulose acetate butyrate and poly-l-lactic acid. The extent of BC attachment to the natural fibers was attributed to the formation of hydrogen bonds at respective interfaces. Study of mechanical properties of natural fibers showed, while BC did not significantly influence sisal fibers, it markedly affected the strength of hemp fibers.

Fortea-Verdejo et al. [164], used bacterial cellulose, nanofibrilated cellulose and pulp fibers to reinforce a nonwoven mat made of flax fibers. Flax fibers were soaked overnight in 10%, 20%, and 30% suspensions of BC, CNF and pulp respectively. Samples were prepared using single step and layer-by-layer papermaking filtration processes where the latter preform showed higher strengths. Both BC and CNF were great binders for loose flax fibers and induced low porosity and higher packing, however pulp was not as effective due to its lower surface area and larger particle size.

Juntaro et al. [76], applied BC on the surface of sisal and hemp natural fibers to compatibilize them with nonpolar polymers. The modified natural fibers were then used to make a composite with cellulose acetate butyrate (CAB) and poly-l-lactic acid (PLLA). An improvement in tensile strength was evident, which was ascribed to the BC binding on the fibers surfaces. Similarly, other researchers coated the sisal natural fibers with BC to produce a denser and reinforced sisal-polylactide acid composite [165].

Lee et al. [166], prepared a similar sisal/bacterial cellulose preform with the difference that fibers were coated by nanocellulose through an overnight soaking process. The dried preform (containing 10 wt % BC) was then mixed with acrylated epoxidised soybean oil (AESO) through vacuum assisted resin injection (VARI) method to produce composites. The resulting sisal-polyAESO and BC-sisal polyAESO composites showed tensile modulus values of 3.2 and 5.6 GPa, respectively, which were markedly greater than that of neat polyAESO (0.4 GPa).

## 8. Nanocellulose in Biomedical Applications

In this section we focus our attention on nanocellulose usages particularly as a binder in the biomedical field. The critical properties of NCs for such applications include but not limited to biocompatibility, tissue-friendliness, non-toxic nature, wound healing properties, antimicrobial effects as well as high binding potential through the available OHs and negative interfacial charges that facilitate the NCs electrostatic adsorption on tissues. The areas that are briefly reviewed in this part are wound dressing, cartilage/bone regeneration, dental repairs, and cancer curing drugs [18].

### 8.1. Wound Dressing

Nanocellulose has been used in wound dressing applications due to its anti-infection features and its ability to increase tensile properties of the scaffolds. Addition of CNC in a collagen-based composite raised its biocompatibility and led to a stable mechanical functionality [167]. Same trend was observed in a following study in which CNCs were added to poly(d,l-lactic-*co*-glycolic acid) (PLGA) and a stronger composite was achieved [168]. Furthermore, CNC was employed in the production of scaffolds to help improving structural strength without any toxicity [169]. Khalid et al. [170] used zinc to load the sheets from bacterial cellulose for burn-healing purposes. The wound healing nature of BC combined with antimicrobial behavior of zinc yielded an efficient burn wound dressing. Using CNF for the threads (filaments) production is another application which can also be considered in biomedical field. The strength of such filaments comes from the intrinsic strength of nanoparticles and the extension of interfibrilar bonding within the filament structure. Recent studies have pointed to promising properties of CNF filaments [171,172,173,174], and their potential usage as suture [175]. Basu et al. coated CNF threads with stem cells and created sutures with positive influence on post-surgery inflammation and wound healing properties [176]. Hakkarainen et al. [177] did a clinical study on the CNF for wound dressing in severely burned patients. They tested CNF dressing in burned skins and reported a successful grafting to the skin donor sites upon the cellulose dehydration. It could be easily attached to the wound bed and remain on the site until the donor site was renewed with no allergic reaction or inflammatory response.

### 8.2. Bone, Cartilage and Dental Restoration

Nanocellulose have also been used in bone regeneration. There are important properties for a better bone regeneration process but facilitating phosphate and calcium deposition are among the most important ones. Saska et al. [178], used BC and collagen to produce a nanocomposite with good binding properties as a suitable option for attaching the collagen to apatite for bone restoration. Results showed that BC-COL-apatite nanocomposite helped the regeneration process with no evidence of cytotoxic, genotoxic, or mutagenic effects. Contrary to other tissues, cartilage has limited ability to regenerate due to its avascular nature. One of the new approaches for repair or regenerate the cartilage is three-dimensional bio-printing which needs a proper bio-ink and specific viscoelastic features. Recent studies on cartilage regeneration proposed a CNF-based bio-ink in which CNF and alginate were used to help bioprinting coupled with human bone marrow–derived stem cells (hBMSCs) and human nasal chondrocytes (hNC) [25,179,180]. The results were promising and proved viability of CNF application as bio-ink for 3D bioprinting of living cells.

For dental applications, materials need to be effectively durable and nanocellulose can be a good choice in the dental field for its reinforcing role. In a work on dental restorative materials [181], the authors used CNF and CNC as additives to dental glass ionomer cement (GIC) matrix and studied the influence of various concentrations of CNF and CNC on the properties of the resulted material. It was reported that while CNF did not incorporate well in the structure, CNC prompted a significant betterment in the mechanical properties of the composite which made it a suitable additive for tooth restoration.

### 8.3. Drug Delivery and Cancer Treatment

Studies have reported that CNF has the potential for improving drug release kinetics. For instance, CNF-based hydrogels were employed as carrier for cancer treatment drugs such as antineoplastic agents [182]. Also, CNF was used to enhance puncture strength of films used in drug delivery for colon-related diseases [183]. The adhesion properties of the CNF were used for anti-metastatic and anti-cancer purposes [184]. Metformin (an anti-cancer drug) was loaded to the surface of nanocellulose (forming a Met-CNF gel structure) to restrict the migration, adhesion and invasion of melonoma cells once implanted around the tumor.

By modifying the surface charge of CNC, it can be employed for the delivery of small interfering RNAs (siRNA) which have therapeutic effects. RNAs in the naked form are not able to be delivered to diseased cell, they also will degrade rapidly however, CNC as a nontoxic, biodegradable unit can facilitate the therapeutic actions by binding to the RNA and performing as a nano-carrier for intracellular delivery [185].

In another study by Plackett et al. [186], CNF, CNC and BC were examined for their drug delivery function and it was revealed that while CNF and CNC could bind well to the water-soluble drugs (via ionic interactions), the function of BC was not as favorable.

## 9. Closing Remarks

Nanocelluloses (cellulose nanofibrils (CNF), cellulose nanocrystals (CNC) and bacterial cellulose, (BC)), represent a natural resource of green and sustainable materials with a magnificent importance today and in the future of nanotechnologies. In the current review we have highlighted some of the most recent advances and contemporary utilization of these nanomaterials in binder-needing areas including wood-based hybrid composites, paper coating process, energy storage devices and biomedical fields as cost-effective and sustainable binder agents. The main emphasis for such applications was on the polymeric nature of cellulose and its inherent ability to self-assemble at interfaces and link to a wide range of materials, especially once within a matrix. This is related to its exceptional structural, chemical and dielectric characteristics that can hardly be found in other polymers. Although development of cellulose-based binders is still in its infancy, it is foreseeable that they take on more adhesive roles in the near future. Undoubtedly, there are also certain challenges on this path for which, in order to overcome them, further fundamental research efforts are needed. For example, adopting novel functionalizing approaches may permit nanocellulose to achieve hierarchical assemblies in various lengths as an essential step towards practical exploitation. In closing, nanocelluloses possess tremendous application potential in binding-related fields and with the aid from multiple scientific disciplines, they may soon become dominant adhesive materials offered by Nature.

## Figures and Tables

**Table 1:** Different cellulose-cellulose interactions relative to molecular distance, data adopted from literature [97,98].

Adhesion Mechanism	Bond Length	Bond Strength (KJ/mol)
Diffusion	<2000 µm	-
Physical entanglement	0.01–1000 µm	-
Van der Waals	0.5–1 nm	8.4–21
Acid-Base Interaction	0.1–0.4 nm	-
Hydrogen bond	0.235–0.27 nm	4.2–188
Covalent bonding	0.15–0.45 nm	147–628
